# Nitriding Titanium by Plasma Ion Implantation: Surface Properties and Initial Osteoblast Cell Response

**DOI:** 10.1590/0103-6440202406111

**Published:** 2024-10-25

**Authors:** Plinio Mendes Senna, Carlos Fernando Mourão, Cindy Goes Dodo, James L. Rutkowski, Altair A. Del Bel Cury

**Affiliations:** 1 Department of Prosthodontics, State University of Rio de Janeiro; 2 Department of Periodontology, Tufts University School of Dental Medicine, Boston; 3 Prosthodontics & Biomaterials Department, Marquette University, Milwaukee; 4 Brooks Rehabilitation College of Healthcare Sciences, Comprehensive Oral Implantology Master’s Degree Program, Jacksonville University, Jacksonville FL; 5 Department of Prosthodontics and Periodontology, Piracicaba Dental School

**Keywords:** dental implants, titanium, osteoblasts, surface treatment

## Abstract

Este estudo in vitro teve como objetivo investigar o efeito da nitretação por implantação iônica de plasma nas propriedades de superfície do titânio e na resposta celular inicial. Discos de titânio grau 4 (12,7 × 2 mm) foram jateados com partículas de óxido de alumínio para criar superfícies moderadamente rugosas. Os discos experimentais (TiN) foram nitrados usando a técnica de implantação iônica de plasma em uma câmara de vácuo. A caracterização da superfície foi realizada usando microscopia confocal a laser, microscopia de força atômica (AFM) e espectroscopia de fotoelétrons de raios X (XPS). A molhabilidade da superfície foi avaliada medindo o ângulo de contato de uma gota séssil usando um goniômetro. Células osteoblásticas humanas foram semeadas nos discos para avaliar a adesão e proliferação celular nos dias 1, 3, 5 e 7 de cultura, usando um ensaio de composto de tetrazólio. A atividade da fosfatase alcalina (ALP) foi medida no dia 7 para avaliar a diferenciação celular. A morfologia celular foi examinada por microscopia eletrônica de varredura (SEM) e microscopia confocal a laser. O grupo TiN exibiu micro-rugosidade similar ao grupo controle; no entanto, apresentou maior densidade de nanostruturas, aumento do conteúdo de nitrogênio e ligeira melhoria na molhabilidade. A proliferação celular e a atividade da ALP foram similares entre os grupos após sete dias de cultura. Em conclusão, a nitretação por implantação iônica de plasma melhora as nanocaracterísticas da superfície e a molhabilidade sem comprometer a biocompatibilidade do titânio, tornando-se uma técnica promissora de modificação de superfície para implantes dentários e ortopédicos.

## Abstract (en)

This *in vitro* study aimed to investigate the effect of plasma ion implantation nitriding on titanium's surface properties and initial cell response. Grade 4 titanium discs (12.7 × 2 mm) were blasted with aluminum oxide particles to create moderately rough surfaces. The experimental discs (TiN) were nitrided using the plasma ion implantation technique in a vacuum chamber. Surface characterization was performed using laser confocal microscopy, atomic force microscopy (AFM), and X-ray photoelectron spectroscopy (XPS). Surface wettability was assessed by measuring the contact angle of a sessile drop using a goniometer. Human osteoblast cells were seeded on the discs to evaluate cell attachment and proliferation at 1, 3, 5, and 7 days of culture using a tetrazolium compound assay. Alkaline phosphatase (ALP) activity was measured at day 7 to assess cell differentiation. Cell morphology was examined by scanning electron microscopy (SEM) and confocal laser scanning microscopy. The TiN group exhibited similar micro-roughness to the control group; however, it displayed a higher density of nanostructures, increased nitrogen content, and slightly improved wettability. Cell proliferation and ALP activity were similar between the groups after seven days of culture. In conclusion, plasma ion implantation nitriding enhances surface nanofeatures and wettability without compromising the biocompatibility of titanium, making it a promising surface modification technique for dental and orthopedic implants.

**Figure:**
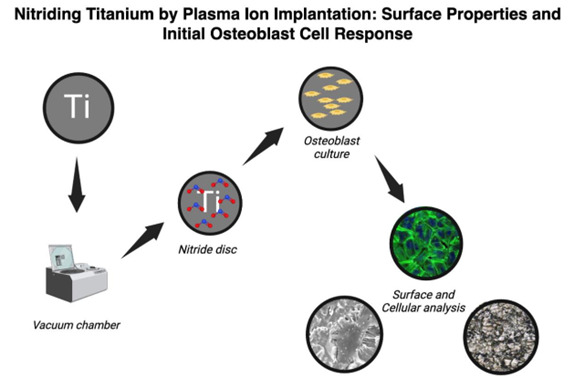

## Introduction

Physical and chemical treatments have been proposed to create rough surfaces on dental implants to improve initial bone formation and support over time ^1^. Most modern dental implant surfaces present surface features with 0.5 to 2 µm average height deviation ^2^. These peaks on the surface may be prone to break and detach during the insertion procedure since titanium has low abrasion resistance ^3^ ^,^ ^4^, explaining why loose titanium particles can be detected in the connective tissue ^5^ ^,^ ^6^. Thus, improving titanium hardness and wear resistance is important since titanium debris in hard and soft tissues is associated with inflammatory response and bone loss ^7^.

Nitriding is a surface treatment commonly used to enhance the wear and corrosion resistance of stainless steel and aluminum alloys. It can be applied to titanium, in which the titanium oxide on the surface is substituted by titanium ^8^. The nitride layer is firmly bonded to the base titanium and does not present mechanical instabilities associated with other additive surface treatments ^8^ ^,^ ^9^. With the higher Vickers hardness of nitrided titanium, the surface demonstrates better abrasion resistance without affecting the biocompatibility of titanium to the bone tissue ^10^ ^,^ ^11^ ^,^ ^12^.

Some nitriding processes are described in the literature to be applied on titanium cp or titanium alloy ^13^ ^,^ ^14^ ^) (^ ^10^ ^,^ ^11^ ^,^ ^12^. Although the hollow cathode technique produced a rougher surface and slightly favored osteoblast differentiation on titanium grade 2 ^13^ ^,^ ^14^, the plasma-based ion implantation produced a smoother surface and higher cell adhesion on titanium grade 5^12^. However the plasma immersion ion implantation was previously presented as a technique to increase roughness at the nanoscale level ^15^ ^,^ ^16^. Therefore, this *in vitro* study aimed to verify the effect of nitrogen in the plasma ion implantation technique on titanium surface properties and biocompatibility.

## Materials and methods

### Titanium discs

Titanium discs (12.7 × 2 mm) were fabricated from a bar of commercially pure grade 4 titanium (Sandinox, Sorocaba, SP, Brazil). Aluminum oxide particles (100 µm diameter) blasted the surface of the titanium discs. The discs were washed in an ultrasonic bath for 20 min in acetone, ethanol, and purified water and dried under vacuum. The experimental discs (TiN) were placed inside a vacuum chamber to be nitrided following the plasma ion implantation technique ^15^. The pressure during the surface treatment was always less than 10^-8^ mbar. First, surface sputtering by argon ions for 10 min at 3 kV was used to remove contaminants and clean the discs. A second bombardment for 30 min at 30 kV in 80% nitrogen/oxygen atmosphere was used for nitriding. The plasma electrode was positioned 10 mm from the surface and the process occurred at 400°C. After, all discs were sterilized by 25 kGy gamma radiation (CBE Embrarad, Jarinu, SP, Brazil).

Three representative sample of each group were used to assess the surface roughness. Laser confocal microscopy (Lext OLS4000; Olympus Corporation, Tokyo, Japan) was used to compute the average height deviation (Sa) and the increased area ratio (Sdr) while atomic force microscopy (Dimension Edge, Veeco Billerica, MA, USA) was used to detect nanostructures on the same sample using a 125 nm cantilever silicon probe in tappin mode (Digital Instruments, Santa Bárbara, EUA). The images were processed with a third order least-squares fit correction to remove errors of tilt and bow (SPIP software v.5.8; Image Metrology A/S, Hørsholm, Denmark). The chemical profile of surface was detected on XPS spectrums (VSW HA-100; VSW Atomtech, Oxfordshire, UK). Each sample was etched with argon ions for three minutes to remove surface impurities.

Surface wettability was determined by the contact angle of a sessile drop (15 µL) of purified water dispensed on the surface of five discs of each group. The contacting angle at the air-liquid-disc intersection was recorded and measured using a goniometer (ramé-Hart 500 advanced; Ramé-hart Instrument Co., Succasunna, NJ, USA). In addition, the surface energy was determined using the Lifshitz-van der Waals/acid-base method ^17^, in which the cosine of the contact angle of water and two extra liquids: bromonaphthalene and formamide (Sigma-Aldrich Corp., St. Louis, MO, USA) were used on extra five discs for each liquid ^18^.

### Cell response analysis

Human osteoblast cells (SAOS-2; ATCC, VA, USA) were used to assess cell proliferation and differentiation. Cells were propagated in McCoy 5A medium supplemented with 15% fetal bovine serum and 1% penicillin-streptomycin (Pen Strep; Gibco Life Technologies, NY, USA). Freshly fed subconfluent cells were harvested, and 2×10^4^ cells were let to adhere on the surface of the discs placed in 24-well tissue plates (TPP, Switzerland). After 3 to 6 hours of incubation, three gentle medium changes washed the non-adherent cells away.

Cell proliferation was evaluated after 1, 3, 5, and 7 days of incubation. After each time point, the discs were washed by medium change and a tetrazolium compound [3-(4,5-dimethylthiazol-2-yl)-5-(3-carboxymethoxyphenyl)-2-(4-sulfophenyl)-2H-tetrazolium] was added to each well (CellTiter 96^®^ AQueous One Solution Cell Proliferation (MTS); Promega, WI, USA). After four hours of incubation at 37°C, all medium was collected, and an aliquot of 100 µl was used to read the absorbance at 490 nm (Multiskan Spectrum Microplate Spectrophotometer, Thermo Fisher Scientific Inc., MA, USA). Six disks from each group were measured at each time point.

At day 7, alkaline phosphatase (ALP) activity was measured to evaluate differentiation using a commercial kit (Labtest Diagnostica SA, Belo Horizonte, MG, Brazil), which detects the release of thymolphthalein from thymolphthalein monophosphate. The cells were collected after trypsin-EDTA treatment and resuspended in 1 ml of DPBS to be lysed by ultrasound (7 W/ 2 min). After, 50 µl of cell lysates were added to the kit, and the absorbance was measured at 590 nm. ALP activity was expressed as U/L. Six disks from each group were measured.

After seven days of culture one disc of each group was selected to cells visualization. The cells were washed by medium change, fixed using paraformaldehyde 4% for 10 minutes, permeabilized with 0.1% triton X-100 in PBS, and washed with PBS. Next, the cells were serially dehydrated in alcohol to be visualized by scanning electron microscopy (model JSM 5600LV; JEOL, Peabody, MA, USA) To be evaluated by confocal laser scanning microscopy (TCS SP5; Leica Microsystems CMS GmbH, Mannheim, German), after washing with PBS, the cells were stained with 1:200 Alexa Fluor 488 phalloidin (Molecular Probes^®^, Life Technologies, USA) in PBS for 1 hours and 1:200 1,5-bis{[2-(di-methylamino)ethyl]amino}a8-dihydroxyanthracene-9,10-dione (DRAQ5, Cell Signalling Technology, MA, USA) for 10 min. The acquired images were processed using Adobe Photoshop software (Adobe Systems, CA, USA).

### Statistical analysis

After checking normality by the Kolmogorov-Smirnov test, the surfaces were compared by the *t*-test with a significance level fixed at 5% (SPSS Statistics 20; IBM Corporation, Armonk, New York, USA).

## Results

**Figure 1:**
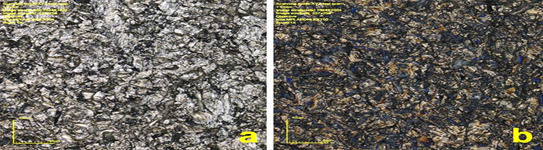
The surface of Ti (a) and TiN discs (b) exhibited similar roughness despite the golden aspect of the TiN surface.

**Figure 2:**
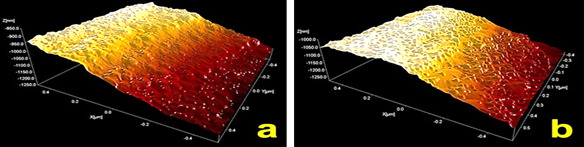
AFM images of Ti (a) and TiN (b) discs.

Figure 1 shows the surface morphology of both groups. Microroughness analyses revealed similar (p > 0.05) Sa values of 1.00 ± 0.04 μm (Ti) and 0.99 ± 0.04 μm (TiN). Similar values were also identified for the increase in surface area (p > 0.05), Sdr values of 34.2 ± 2.7 % for Ti and 34.4 ± 1.7 % for TiN. However, in the AFM analysis, the TiN surface exhibited a higher concentration of nanostructures than the Ti surface (Figure 2).

**Figure 3:**
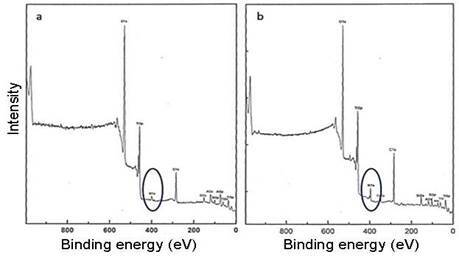
XPS diffractograms of Ti (a) and TiN (b) surfaces.

The XPS result showed a significant N1s peak (∼400 eV), corresponding to an increased amount of nitrogen in the TiN surface (Figure 3). It was assumed that the bonding between Ti and N had occurred until 150 nm depth. The deconvolution of Ti2p and O1s peaks demonstrates no structural change in the surface oxide layer. In addition, small peaks relative to aluminum and silicon on both surfaces indicate the presence of particles from the blasting procedure. Despite of increased C1s peak in TiN surface, better wettability with higher polar interaction (p < 0.05) than Ti surface (Table 1) was identified. 

**Figure 4:**
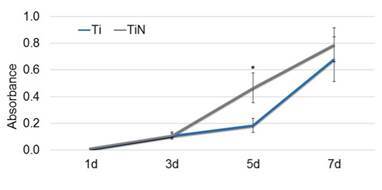
Cell proliferation after 1, 3, 5, and 7 days of culture.

**Figure 5:**
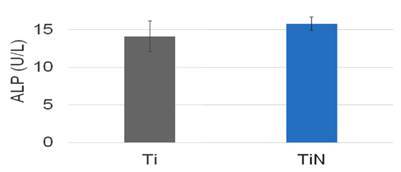
Alkaline phosphatase levels after seven days of culture of SAOS-2 cells on Ti and TiN discs. No significant difference was seen (*p* > 0.05, *t*-test).

**Figure 6:**
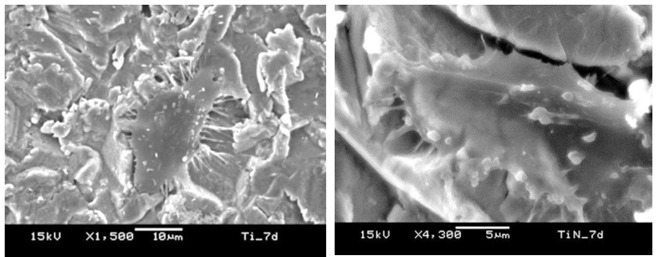
SEM revealed dendritic projections and small filopodia in SAOS-2 cells adhered on both Ti (a) and TiN surfaces (b).

**Figure 7:**
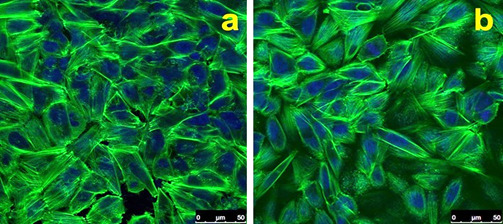
Confocal images showed a similar distribution of cells on Ti (a) and Tin (b) discs. In green is the actin stained by phalloidin, and in blue is the core stained by the DRAQ5 dye.

Cell analyses revealed similar cell attachment and proliferation up to seven days of culture (Figure 4) with a low production of ALP (Figure 5). This indicates the same stage of cell differentiation in both groups. Microscopy images revealed lamellipodia and filopodia among the SAOS-2 cells adhered on surfaces (Figure 6) with similar cell density (Figure 7).

**Table 1:** Wettability and surface energy of Ti and TiN surfaces (mean ± s.d.).

	Water contact angle	Surface energy		
		Polar component	Dispersive component	Total energy
TiN	75.6 ± 3.1° *	10.4 ± 5.0 *	25.1 ± 2.8	32.0 ± 2.3
Ti	80.4 ± 4.6°	6.3 ± 5.0	26.3 ± 1.2	31.1 ± 2.9

* Indicates significant surface differences (p < 0.05, t-test).

* Indicates significant surface differences (p < 0.05, t-test).

## Discussion

The present study evaluated the impact of nitrogen in the plasma ion implantation method on titanium surface properties and biocompatibility and demonstrated that there was an increase of surface nanofeatures without affecting titanium biocompatibility. The study reveals that the nitrided titanium surface has a higher nanostructure density than the control titanium surface while maintaining similar microroughness parameters. In addition, the nitriding process improved the surface wettability by increased polar interactions. 

Different methods have been tested to implement nitrogen on the surface. A previous study, which used the cathodic cage technique to nitride titanium, showed a rougher surface after treatment than the control group ^13^ ^,^ ^14^. In the same way, the present study identified higher nanostructures on surface despite no effect on microroughness. Conversely, a previous study produced a smoother surface with similar nitrogen plasma treatment ^12^. The different result can be explained by other plasma parameters, such as the temperature and the voltage, and the fact they applied on titanium grade 5 instead of grade 4.

Although surface morphology differences, the surface wettability is also important to cell adhesion ^19^. Hydrophilic surfaces generally offer better conditions for cell adhesion than hydrophobic surfaces. In the present study, the ion implantation technique produced a surface with slightly better wettability that did not reflect better cell adhesion. In contrast to Hotchkiss and colleagues that showed more carbon (C1s) on hydrophobic surfaces ^20^, the present study demonstrated increased C1s peak in the XPS analysis and increased wettability. Previous studies also reported increased wettability of the nitrided titanium surface ^12^ ^,^ ^13^ ^,^ ^14^.

In the present study, no significant difference in cell attachment and proliferation was seen between the groups at the time length studied. Moreover, similar patterns of cell morphology were identified on the nitrided and control titanium surfaces. These findings indicate that the nitrided layer is as biocompatible as the titanium surface to the cells ^21^. In contrast, previous studies reported higher cell adhesion on the nitrided surface ^12^ ^,^ ^13^ ^,^ ^14^. A delicate balance between cell proliferation and differentiation is essential for an adequate tissue response ^14^. To track cell differentiation, the present study evaluated ALP activity, a key osteoblast marker. Usually, the ALP activity increases over time; however, in the present study, the cells were undifferentiated in both groups, with low production of ALP, which can be expected for SAOS-2 cells derived from the osteosarcoma phenotype.

Attention is given to the surface characteristics to determine which properties affect osseointegration. In this way, the nitriding treatment by plasma ion immersion would be an interesting possibility since it is a clean and low-cost physical treatment that requires short treatment time to produce controlled surface modification ^13^. In addition, plasma nitriding on surface topography was limited to modify the nano-structure density without great changes in the structure of the titanium oxide layer. Although positive *in vitro* results ^12^ ^,^ ^13^ ^,^ ^14^, there is limited data of *in vivo* bone response ^22^. Thus, further *in vivo* studies are necessary to evaluate nitrided titanium implants' long-term osseointegration and stability.

## Conclusion

Nitriding by plasma ion immersion can be used to improve wettability and the presence of nanostructures on titanium surface without compromising their biocompatibility.
